# Refining a taxonomy for guideline implementation: results of an exercise in abstract classification

**DOI:** 10.1186/1748-5908-8-32

**Published:** 2013-03-15

**Authors:** Danielle Mazza, Phillip Bairstow, Heather Buchan, Samantha Paubrey Chakraborty, Oliver Van Hecke, Cathy Grech, Ilkka Kunnamo

**Affiliations:** 1 Department of General Practice, School of Primary Health Care, Faculty of Medicine Nursing and Health Sciences, Monash University, Ferntree Gully Rd, Notting Hill, Victoria, Australia; 2 Diagnostic Imaging Pathways, Royal Perth Hospital, Wellington St, Perth, WA, Australia; 3 Australian Commission on Safety and Quality in Health Care, Oxford St, Darlinghurst, NSW, Australia; 4 The Finnish Medical Society Duodecim, Helsinki, Finland

**Keywords:** Guideline implementation, Taxonomy, Interventions, EPOC checklist, Ontology

## Abstract

**Background:**

To better understand the efficacy of various implementation strategies, improved methods for describing and classifying the nature of these strategies are urgently required. The aim of this study was to develop and pilot the feasibility of a taxonomy to classify the nature and content of implementation strategies.

**Methods:**

A draft implementation taxonomy was developed based on the Cochrane Effective Practice and Organisation of Care (EPOC) data collection checklist. The draft taxonomy had four domains (professional, financial, organisational and regulatory) covering 49 distinct strategies. We piloted the draft taxonomy by using it to classify the implementation strategies described in the conference abstracts of the implementation stream of the 2010 Guideline International Network Conference. Five authors classified the strategies in each abstract individually. Final categorisation was then carried out in a face-to-face consensus meeting involving three authors.

**Results:**

The implementation strategies described in 71 conference abstracts were classified. Approximately 15.5% of abstracts utilised strategies that could not be categorised using the draft taxonomy. Of those strategies that could be categorised, the majority were professionally focused (57%). A total of 41% of projects used only one implementation strategy, with 29% using two and 31% three or more. The three most commonly used strategies were changes in quality assurance, quality improvement and/or performance measurement systems, changes in information and communication technology, and distribution of guideline materials (via hard-copy, audio-visual and/or electronic means).

**Conclusions:**

Further refinement of the draft taxonomy is required to provide hierarchical dimensions and granularity, particularly in the areas of patient-focused interventions, those concerned with audit and feedback and quality improvement, and electronic forms of implementation, including electronic decision support.

## Background

Effective implementation strategies are necessary for improving the uptake and use of clinical practice guidelines by the intended guideline audience [1,2]. We define ‘implementation strategy’ as a purposeful procedure to achieve clinical practice compliance with a guideline recommendation. There are, however, a number of barriers to consider when selecting which implementation strategies to pursue; chief among these is the dearth of systematic evidence to support effective guideline implementation, and a lack of a purposeful, rigorously developed framework for the planning, implementing and reporting of intervention strategies [3].

The lack of such a framework inhibits the shared understanding of the exact nature of specific implementation strategies, which makes it difficult to design and conduct effectiveness studies to investigate differences between strategies [4-6]. This is amplified by a number of factors, such as the variability in reported structure and definition of the nature of the strategies [6], the level of detail used to describe the strategies, lack of specification regarding the intended user of the strategy, and variability in the language used to describe the design of the strategy [4]. Difficulties in assessing the effectiveness of implementation strategies could also be due to variations in the designs of implementation strategies. For example, the effectiveness of audit and feedback strategies in healthcare settings is related to features such as providing correct solution information in the feedback, providing written feedback, and more frequent feedback being incorporated into the feedback strategy [7].

The lack of clarity with regards to the nature of implementation strategies obscures the understanding of study outcomes, making it difficult to correlate findings from a range of data sets or to pool data from several studies in a systematic review to determine the effectiveness of a specific strategy [4]. The lack of an agreed and consistent taxonomy for describing the available strategies in the literature also poses a difficulty for those wishing to publish their findings about the effectiveness of various implementation strategies [8].

### Why develop a taxonomy?

Taxonomies are effective modes of information management that have been used successfully in medicine, business systems, and information technology to describe, classify, and organise items on the basis of shared characteristics [9,10]. A taxonomy of implementation strategies would provide a standardised framework for the consideration of strategies to improve the uptake of evidence into practice. Specifically, it could improve the clarity of definitions of specific implementation strategies, provide researchers with criteria with which to improve the quality of reporting of research, provide those involved in implementation with the tools to find and assess the effectiveness of various strategies, and assist end users to undertake purposefully defined steps to ensure behavioural change [11]. There is a growing body of literature describing implementation strategies [12], and these descriptions can inform a scheme for the categorisation of these strategies [13].

### Value of current reporting systems and structures for reporting implementation research

A number of strategies have been developed to provide authors with publication guidelines for reporting the outcomes of implementation research. While these are widely used, they do not provide the level of detail required for defining and reporting the nature of implementation strategies [14,15]. The most commonly used publication guidelines are the Consolidated Standards Of Reporting Trials (CONSORT) [16], Transparent Reporting of Evaluations with Nonrandomized Designs (TREND) [17], and STrengthening the Reporting of OBservational studies in Epidemiology (STROBE) [18] statements, which have been used to guide the reporting of randomised controlled trials, non-randomised trials, or cohort, case–control, and cross-sectional studies, respectively.

While these guidelines and other statements have been widely used to provide researchers with a concise and practical method to assess and report implementation strategies, there is still a need for improved reporting of implementation strategies and for more sophisticated guidelines for reporting different strategies [6,8]. Some guidelines do not provide sufficient information on the definition or design of individual strategies; instead, there is a reliance on authors to be specific, detailed, and to use terminology that is consistent with other researchers [6,14,15,19]. The Workgroup for Intervention Development and Evaluation Research (WIDER) group have proposed a set of recommendations to journal editors for improving the reporting of behaviour change interventions and their evaluations in accordance with CONSORT statements [20]. While these recommendations are yet to be widely implemented, they could improve the understanding and replication of behaviour change interventions reported in literature.

### Taxonomies in development

Numerous topic-specific taxonomies of implementation strategies have been reported in the literature [21-23]; however, their specificity prevents them from offering a comprehensive view of strategies for effective guideline implementation. Two additional tools that provide broad guidance on implementation strategies are the Intervention Taxonomy (ITAX) [24] and Effective Practice and Organisation of Care (EPOC) Data Collection Checklist [25]. The ITAX provides a comprehensive list of features that make up an implementation strategy (e.g., mode, materials, location, schedule, scripting sensitivity to participant characteristics, intervention characteristics, adaptability, and treatment implementation); however, each of these aspects is addressed separately [24]. In contrast, the EPOC checklist enables considerations of combinations of factors involved in implementation by distinguishing between four domains: professional strategies, organisational strategies, financial strategies, and regulatory strategies [25]. The EPOC checklist was created for the purpose of assisting a group of reviewers to select papers for inclusion in a systematic review [25], rather than for guideline implementers to select the appropriate implementation strategy. Nevertheless, these domains are in line with the intervention categories described in the literature [2,26] and, as such, were used as a starting point to develop the taxonomy described in this paper.

### Aim

The aim of our study was to draft an implementation taxonomy and to pilot its usefulness and feasibility as a tool for classifying implementation strategies.

## Methods

### Development of the taxonomy

The development of the taxonomy occurred after correspondence between two of the authors (IK, PB) about taxonomies that could be applied in the implementation of the Diagnostic Imaging Pathways Application (http://www.imagingpathways.health.wa.gov.au). The EPOC Data Collection checklist was used as a starting point, which led to an interest in a broader approach of using the EPOC checklist items (see EPOC Data Collection Checklist) to classify the abstracts from the 2008 and 2009 Guidelines International Network (G-I-N) Conferences. G-I-N is a network of guideline organisations, implementers, end-users, researchers, students, and other stakeholders who are working towards improving the efficiency and effectiveness of evidence-based guideline development, adaptation, dissemination and implementation (http://www.g-i-n.net/). The classification of abstracts using the EPOC checklist led to some difficulties, which included difficulty in relating the prose of the checklist items to guideline implementation, repeated efforts to read and re-read checklist items because of the length of the definition and inclusion criteria, some checklist items were irrelevant to guideline implementation, lack of a logical order with checklist items within major categories, checklist items inappropriately categorised, and the absence of checklist items that describe some well-established methods of guideline implementation. Consequently, a revised taxonomy was drafted based on the EPOC checklist, which included 49 items listed under four broad domains: professional, financial, organisational, and regulatory.

Summary of items from the EPOC Data Collection Checklist in the category of ‘Type of intervention’ (excluding definitions and inclusion criteria)

Types of interventions

Professional interventions

Distribution of educational materials

Educational meetings

Local consensus processes

Educational outreach visits

Local opinion leaders

Patient mediated interventions

Audit and feedback

Reminders

Marketing

Mass media

Other

Financial interventions

Provider interventions

Fee-for-service

Prepaid

Capitation

Provider salaried service

Prospective payment

Provider incentives

Institutional incentives

Provider grant/allowance

Institution grant/allowance

Provider penalty

Institution penalty

Formulary

Other

Patient interventions

Premium

Co-payment

User-fee

Patient incentives

Patient grant/allowance

Patient penalty

Other

Organisational interventions

Provider orientated interventions

Revision of professional roles

Clinical multidisciplinary teams

Formal integration of services

Skill mix changes

Continuity of care

Satisfaction of providers

Communication and case discussion

Other

Patient orientated interventions

Mail order pharmacies

Presence and functioning of adequate mechanisms for dealing with patients’ suggestions and complaints

Consumer participation in governance of health care organisation

Other

Structural interventions

Changes in the setting/site of service delivery

Changes in physical structure, facilities and equipment

Changes in medical records systems

Changes in scope and nature of benefits and services

Presence and organisation of quality monitoring mechanisms

Ownership, accreditation, and affiliation status of hospitals and other facilities

Staff organisation

Other

Regulatory interventions

Changes in medical liability

Management of patient complaints

Peer review

Licensure

Other

### Pilot and feasibility testing of the updated taxonomy

To test its utility and comprehensiveness, we applied our draft taxonomy to a convenience sample of conference abstracts from the implementation stream of the 2010 G-I-N Conference. Abstracts were independently classified by five reviewers (DM, IK, PB, OVH, CG) using the draft taxonomy.

Reviewers utilised a classification template developed by HB that assessed whether the abstract referred to a research study, whether the study described strategies used to implement a guideline, which of the strategies in the draft taxonomy could be used to categorise the reported interventions, and whether the study described a method for measuring the impact/outcome of implementation. Three of the reviewers (DM, OVH, CG) then met face-to-face to establish a consensus regarding the exact implementation strategies used in the relevant abstracts and to highlight areas of the taxonomy requiring further refinement.

The draft taxonomy and outcomes from the pilot study were presented to a self-selected group of interested participants who attended a G-I-N meeting workshop in 2011, and feedback was sought from this group about the purpose, design and content of the draft taxonomy.

## Results

### Components of the taxonomy

Our draft taxonomy comprises four domains of intervention types: professional, financial, organizational, and regulatory. These domains were preserved from the original EPOC checklist. Within the taxonomy, 49 implementation strategies are described: 15 strategies targeting health professionals; 12 strategies involving financial incentives for guideline implementers (eight) and for patients (four); 18 involving organisational strategies at an implementer level (six), at a patient level (three), and at a structural level (nine); and 4 strategies involving structural change. The draft taxonomy is provided as Additional File 1.

### Results of pilot testing

The following findings relate to the application of the updated draft taxonomy to the abstracts from the implementation stream of the G-I-N 2010 Conference.

### Overview of abstracts

A total of 85 abstracts were available for inclusion in the pilot study. Of these, 71 abstracts included a description of guideline implementation strategies and were, therefore, used in the study. Fifty-six abstracts described original research studies involving guideline implementation, and 15 abstracts described non-research studies (projects) involving guideline implementation.

### Number of strategies described in each study

**Figure 1:**
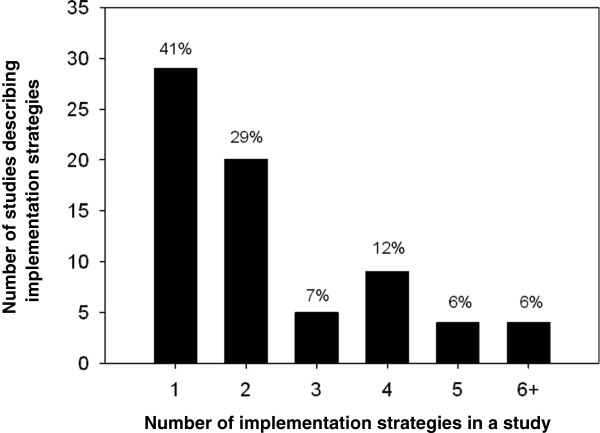
Number of interventions described in each study (n = 71).

A total of 170 implementation strategies were described in the 71 abstracts. Figure 1 shows the variability in the number of guideline implementation strategies described in abstracts. The majority of abstracts (60%) reported using two or more strategies in their study. The most common strategies used were single-strategy (41%) or a combination of two strategies (29%).

### Use of the domains of the taxonomy

**Figure 2:**
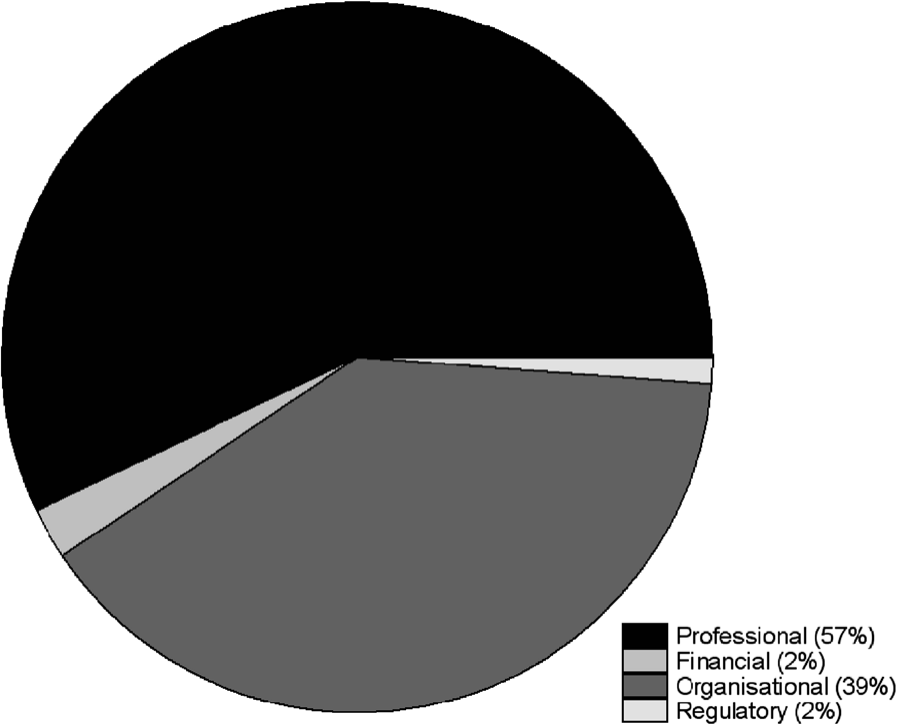
Distribution of interventions by domains of the taxonomy (n = 170).

All four domains of the taxonomy were utilised in the assessment of the abstracts. However, the implementation strategies were spread disproportionately across the four domains (Figure 2). Strategies focusing on ‘professional’ aspects were employed most often, followed by ‘organisational’ strategies, while ‘financial’ and ‘regulatory’ strategies were utilised minimally in the activities described by the abstracts.

### Use of strategies within domains of the taxonomy

The most commonly used strategies are presented in Table 1. Similar to the characterisation of strategies by domain, all of the top 10 strategies for guideline implementation referred to either professional (seven strategies) or organisational changes (three strategies). The frequency at which each implementation strategy was reported is presented in Table 2. Of the list comprising 49 possible strategies, 18 of these were not utilised at all by researchers presenting their work in the implementation stream of the G-I-N meeting in 2010. A number of other strategies involving the financial and regulatory domains or strategies involving patients were also minimally utilised.

**Table 1:** Hierarchy of top 10 most used strategies

**Strategy** (**Domain**)	**Number of abstracts (%)**
Change in quality assurance, quality improvement and/or performance measurement systems (Organisational)	23 (*32*)
Change in information & communication technology (Organisational)	21 (*30*)
Distribute guideline materials (Professional)	17 (*24*)
Identify barriers to guideline implementation (Professional)	15 (*21*)
Educate groups of health care professionals (Professional)	15 (*21*)
Creation of an implementation team (Organisational)	10 (*14*)
Other (Professional)	10 (*14*)
Feedback guideline compliance data and information (Professional)	9 (*13*)
Educate individual health care professionals (Professional)	6 (*8*)
Provide reminders (Professional)	6 (*8*)

**Table 2:** Number of abstracts identifying each implementation strategy

**Item No.**	**Description**	**Number of abstracts**
1 Professional Strategies		
1.2	Distribute guideline materials	17
1.1	Identify barriers to guideline implementation	15
1.6	Educate groups of health care professionals	15
1.15	Other	10
1.11	Feedback guideline compliance data and information	9
1.5	Educate individual health care professionals	6
1.9	Provide reminders	6
1.7	Recruit an opinion leader	5
1.3	Advertise guideline materials	3
1.4	Present guideline materials at meetings	3
1.8	Achieve consensus	3
1.1	Provide alerts	3
1.12	Feedback data and information about	1
1.13	Feedback data and information from patients	1
1.14	Feedback information from health care professionals	0
2.1 Financial Strategies (implementer)		
2.1.3	Grant or allowance provided to a health care professional	2
2.1.2	Incentive applicable available to the institution	1
2.1.4	Grant or allowance provided to the institution	1
2.1.1	Incentive applicable to a health care professional	0
2.1.5	Penalty applicable to a health care professional	0
2.1.6	Penalty applicable to the institution	0
2.1.7	Change in reimbursement	0
2.1.8	Other	0
2.2 Financial Strategies (patient)		
2.2.1	Incentive applicable to a patient	0
2.2.2	Grant or allowance provided to a patient	0
2.2.3	Penalty applicable to a patient	0
2.2.4	Other	0
3.1 Organisational Strategies (implementer)		
3.1.3	Creation of an implementation team	10
3.1.1	Additional human resources	3
3.1.2	Reallocated roles	1
3.1.4	Communication between distant health professionals	1
3.1.5	Improved health care professional satisfaction	0
3.1.6	Other	0
3.2 Organisational Strategies (patient)		
3.2.1	Consumer participation	1
3.2.2	Consumer feedback, suggestions and complaints	1
3.2.3	Other	1
3.3 Organisational Strategies (structure)		
3.3.5	Change in quality assurance, quality improvement and/or performance measurement systems Change in the method	23
3.3.4	Change in information & communication technology	21
3.3.6	Change in the method	3
3.3.1	Change in organizational structure	1
3.3.7	Change in the integration of services	1
3.3.2	Change to the setting or site	0
3.3.3	Change in the physical structure, facilities or equipment	0
3.3.8	Change in risk management provisions	0
3.3.9	Other	0
4 Regulatory Strategies		
4.1	Change in legislation or regulation	1
4.4	Other	1
4.2	Change in the ownership or affiliation	0
4.3	Change in licensing, credentialing or accreditation	0

### Appropriateness of nomenclature used in the taxonomy

The distribution of reported implementation strategies among the domains of the taxonomy are presented in Table 2. Of note was the inability of the taxonomy to accurately classify strategies described in a large number of abstracts in this study beyond that of the broad domains. A total of 11 of the 71 abstracts (15.5%) utilised strategies that could not be categorised using the draft taxonomy. Ten of these were ‘other’ professional strategies, which included ‘a bundle of care approach,’ ‘development of resources,’ and ‘algorithm,’ while one referred to an ‘other’ organisational structure-related strategy.

## Discussion

Using the EPOC checklist as a starting point to code strategies described in abstracts from the 2008 and 2009 G-I-N Conferences, we drafted an implementation taxonomy, which was subsequently used to classify implementation strategies described in the abstracts from the implementation stream of the 2010 G-I-N Conference. Although all four domains (professional, financial, organisation, and regulatory) were utilised in the assessment of the abstracts, our draft taxonomy proved inadequate when attempting to further classify some of the implementation strategies using the subgroups within these domains.

### Content-related issues

All of the domains in our draft taxonomy were successfully applied to the abstracts, which supports their usefulness as broad categorisations. However, further classification of the abstracts using the categories within each domain yielded a high number of strategies that were classified as ‘other’. Use of the ‘other’ category is likely due to the level of detail available in abstracts, which could be overcome by reviewing a full-text article. However, it may also point to the need for better granularity within the taxonomy to allow more accurate classification of strategies. Further investigation is required to identify and understand the types of strategies that have not been included in the current version of the EPOC taxonomy.

The language and clarity of our draft taxonomy were assessed using feedback received from attendees at a workshop on the taxonomy, which was held at the G-I-N 2011 meeting. In general, the taxonomy was found to be sufficiently detailed to enable classification. However, in some instances, items were found to be less explicit. For example, the strategy ‘identify barriers’ does not specify the type of barrier or the method used for identification.

### Design-related issues

A number of design-related issues were also identified by the authors when the draft taxonomy was used to classify the abstracts. The level of detail included in each subgroup of the taxonomy was, in some instances, not sufficiently detailed to distinguish differences between two items on the checklist. For example, problems arose when trying to differentiate reminders and recalls from other forms of electronic implementation and when differentiating audit and feedback from other quality improvement activities. These issues support the need for improving the granularity of the taxonomy.

The focus on interventions for health professionals in this taxonomy also overlooks other groups of individuals, such as patients and key decision makers, who play a critical role in the health system. There is an increasing recognition of the benefits of patient involvement in healthcare, particularly in patient-centred health care and shared decision-making [27-30]. As such, it is important to consider implementation strategies aimed at improving the role of consumers in the implementation of guidelines. For example, the draft taxonomy, at present, provides only seven possibilities for patient-directed strategies, which fit within the ‘Financial’ or ‘Structural’ domains, while a substantial portion of the taxonomy describes strategies for health professionals. The scarcity of consumer-related implementation strategies in the EPOC taxonomy may be supplemented with strategies identified by the EPOC Consumers and Communication Group and published via the Cochrane Library, as well as other current literature.

In addition to health professionals and patients, knowledge translation in health care involves other groups such as policy makers, government officials, membership bodies, and non-government organisations. The present structure of the taxonomy does not permit consideration of the specific roles of these groups within the healthcare system and, as such, would require revision to ensure that implementation strategies aimed at behaviour change in a range of participants within the healthcare system are included in the framework.

### Multifaceted strategies

Following the process of classifying implementation strategies using our draft taxonomy, we found that the majority of abstracts reported the use of two or more strategies. The use of multiple strategies by some guideline implementers (with some abstracts describing six or more activities) suggests that implementers are cautious about confining themselves to a select number of strategies. Alternatively, it suggests that the use of multiple strategies is assumed to be more effective because it addresses more barriers [31]. Nevertheless, the use of multiple strategies highlights the difficulty in determining causality and, in turn, the effectiveness of individual implementation strategies when more than one is used.

It is also currently unclear how guideline implementers select the number of strategies required to facilitate the implementation of a guideline. While a number of studies have demonstrated improved patient outcomes after multifaceted interventions [32], more evidence is required to demonstrate the optimal number or combination of strategies for guideline implementation and the circumstances under which the number of strategies is most beneficial.

### Professional and organisational strategies

The results of our study showed that the commonly used implementation strategies described within the abstracts were either professional or organisational. These results may reflect the fact that: there is more evidence to support the use of these strategies; these strategies are more accessible to guideline implementers in general; or these strategies are more accessible to the cohort of presenters at the G-I-N Conference who are likely to be researchers [33]. Conversely, the low utilisation of financial and regulatory interventions in the selected abstracts may reflect the fact that research involving these implementation strategies is not being sufficiently reported in scientific fora. Low usage of financial and regulatory interventions may also reflect the capabilities of the authors of these research papers, who may be clinicians or academics working in healthcare settings, to access these strategies.

### Limitations of the study

We identified a few limitations in our study. The review of abstracts provided a lower level of detail compared to that of a full-text article. In some instances, this lower level of detail made it difficult to accurately classify the implementation strategies described in the abstract. In these instances, it is difficult to ascertain if the inability to classify a strategy is due to the detail and accuracy of the abstract or the draft taxonomy itself. Nevertheless, abstracts are usually the first ‘screening point’ for published papers and, therefore, it is important that abstracts, as well as full-text articles, include a sufficient level of detail to inform the reader about strategies that were used.

Another limitation of this study is the use of abstracts from the G-I-N Conference. Applying the taxonomy on these abstracts may yield optimistic depictions of guideline implementation, because the participants at this conference are likely to be more conscientious about the implementation of guidelines than people in the broader academic or healthcare community. Nevertheless, these abstracts provide a good basis for testing our draft taxonomy as they are screened and selected by an expert panel and, therefore, the reporting of details in these abstracts is likely to be of a higher standard than those published in the wider literature. Further development of our draft taxonomy will include its application on full-text articles, which would greatly enhance its utility, validity, and relevance.

### Next steps

This first draft of the taxonomy utilised a flat structure to create a blueprint of a potential hierarchical format of the taxonomy. Knowledge translation can, however, occur in three dimensions: linear, cyclic, and multi-dimensional [34], and guideline implementation requires a complex assessment of considerations of different interventions, levels of intervention, target groups, and contextual differences [8,11]. Our draft taxonomy could be altered so that strategies can be classified across many dimensions or elements. For example, a patient-based implementation tool that is also information technology-based and gives feedback to a doctor may be viewed as having three dimensions. This multi-dimensional approach to implementation could be supported by expanding the taxonomy into a multi-dimensional framework or an ontology to aid guideline developers and implementers to consider the relationships between components that make up implementation strategies [35,36]. A taxonomy of interventions could also be used in conjunction with an appropriate reporting structure, as it complements current reporting guidelines by providing definitions for a broad categorisation of intervention strategies.

Behavioural change techniques may also intersect with guideline implementation strategies. It is therefore important to consider the reliability of language and coding for implementation strategies used within the guideline, as these could contribute to guideline implementation but could also be classified as behavioural change [37]. Future iterations of the taxonomy should, therefore, consider whether behavioural change techniques need to be differentiated from guideline implementation strategies and if so, how this can be done.

### Implications

Further development of our draft implementation taxonomy should include a review of other taxonomies for supplementary items that may be relevant to our current taxonomy and the inclusion of strategies that were classified as ‘other’ in the current draft. This could result in a more useful tool for guideline implementers and researchers. A discussion of the strengths and weaknesses of our taxonomy in comparison to other published taxonomies, including those related to specific healthcare settings [38], would also increase its validity. Specific revisions to the taxonomy will need to consider the expansion of professional strategies to include the range of professionals, consumers, and other groups involved in implementation and the development of a hierarchical structure or possibly a multi-dimensional ontological framework. Further investigation is also required as to which strategies should be included in the ‘other’ category; and the feasibility study needs to be replicated using published research papers instead of abstracts. Input from other implementation science experts should also be sought to strengthen the validity of our draft taxonomy. Nevertheless, we believe that our draft taxonomy is likely to be applicable to other settings, given that the development process involved the use of abstracts from the G-I-N Conference, which attracts implementations researchers from various settings.

While there continue to be issues relating to the reporting of interventions (e.g., the rigour of design and reporting of research versus evaluation), the availability of a taxonomy moves us one step closer to providing structure for reporting and assessing implementation strategies. Guideline implementers and researchers would also benefit from the availability of a well-indexed database of implementation studies, which would facilitate the search for and identification of suitable implementation strategies. One such example is the Health Evidence Database Classification (http://www.mcmasterhealthforum.org/healthsystemsevidence-en), which is a database that contains research evidence about governance, finance, and delivery arrangements of healthcare systems, as well as implementation strategies. Improving the accuracy and granularity of the MeSH vocabulary [39] for describing implementation research would also facilitate the indexing of implementation strategies.

However, the use of a taxonomy in research reports does not, by any means, reduce the need to describe the implementation strategy in detail. Sufficient information is still needed to enable implementers to replicate the strategy in other locations and settings, which is often not the case even in treatment trials [40]. Recommendations and checklists such as the Guideline for Reporting EBP Educational interventions and Teaching (GREET) statement, which is being developed for educational interventions, will be helpful [41].

## Conclusions

Further refinement of the draft taxonomy is required to provide hierarchical dimensions and granularity, particularly in the areas of patient-focused interventions, those concerned with audit and feedback, quality improvement, electronic forms of implementation, and reminders and alerts which might arise from electronic decision support. Also, as several groups and organizations develop implementation taxonomies, better collaboration between them is urgently needed. Groups such as G-I-N and the EPOC Review Group could provide leadership in this area and facilitate collaborations to produce an implementation taxonomy that has been reached by consensus and applies to all areas of implementation research.

## Abbreviations

CONSORT: Consolidated Standards Of Reporting Trials; TREND: Transparent Reporting of Evaluations with Nonrandomized Designs; STROBE: Strengthening the Reporting of OBservational studies in Epidemiology; SQUIRE: Standards for Quality Improvement Reporting Excellence; RE-AIM: Reach Effectiveness Adoption Implementation Maintenance; ITAX: Intervention Taxonomy; EPOC: Effective Practice and Organisation of Care; G-I-N: Guidelines International Network.

## Competing interests

Heather Buchan is a member of the Implementation Science Editorial Board.

## Authors’ contributions

DM classified the abstracts using the taxonomy, led the consensus process, the writing of the paper, the analysis of the results and developed the discussion themes; PB was involved in drafting the taxonomy and classification of the abstracts; HB was involved in drafting the taxonomy and initial classification of the 2008 and 2009 abstracts in order to develop the eventual template used to classify the abstracts; SC drafted the paper; OVH and CG classified the abstracts and were involved in the consensus process; IK was involved in designing the project, drafting the taxonomy and classification of the abstracts. All authors contributed to the editing of the paper and approved the final version.

## Supplementary Material

Additional file 1 Strategies for implementation (strategies for achieving guideline implementation and compliance). Click here for file
