# Supplementary material for: Refining a taxonomy for guideline implementation: results of an exercise in abstract classification
Source: Implement Sci. 2013 Mar 15;8:32. doi: 10.1186/1748-5908-8-32 (PMC3606141; doi:10.1186/1748-5908-8-32)
Supplement: Additional file 1 — Strategies for implementation (strategies for achieving guideline implementation and compliance). [file 1748-5908-8-32-S1.doc]

# Additional File 1

**Strategies for implementation (strategies for achieving guideline implementation and compliance)**

# 1 Professional

1.1 Identify barriers to guideline implementation (including any activity aimed at identifying reasons why compliance with a guideline might not be achieved, to assist in planning strategies)

1.2 Distribute guideline materials (via hard-copy, audio-visual and/or electronic means)

1.3 Advertise guideline materials (including advertising via any medium, targeted advertising, personal interviews, group discussions aimed at raising awareness)

1.4 Present guideline materials at meetings (including conferences, lectures, workshops or traineeships)

1.5 Educate individual health care professionals about the intent and benefit of complying with a guideline

1.6 Educate groups of health care professionals about the intent and benefit of complying with a guideline

1.7 Recruit an opinion leader who recommends the implementation of a guideline (health care professionals must recognize the authority of the opinion leader in regard to the guideline)

1.8 Achieve consensus among health care professionals that the guideline is appropriate for implementation (there must be data that measures consensus)

1.9 Provide reminders to individual health care professionals or groups about the intent and benefit of complying with a guideline (via any means)

1.10 Provide alerts to individual health care professionals or groups when clinical practice deviates from a guideline (via any means)

1.11 Feedback guideline compliance data and information to individual health care professionals or groups to improve compliance (including feedback on non-compliance with a guideline)

1.12 Feedback data and information about patients to individual health care professionals or groups to improve compliance (including clinical outcome data and information, and patient self-assessments)

1.13 Feedback data and information from patients to individual health care professionals or groups to improve compliance (including any new process that involves direct communication between a patient and health care professionals that improves implementation)

1.14 Feedback information from health care professionals to individuals or groups to improve compliance (including personal testimony about the experience of implementing a guideline)

1.15 Other

# 2 Financial

### 2.1 Health care professionals

2.1.1 Incentive applicable to a health care professional (a health care professional may receive a direct or indirect financial reward or benefit for complying with a guideline)

2.1.2 Incentive applicable available to the institution (the institution or a group of health care professionals may receive a direct or indirect financial reward or benefit for complying with a guideline)

2.1.3 Grant or allowance provided to a health care professional (a health care professional received direct or indirect financial reward or benefit but not tied to being compliant)

2.1.4 Grant or allowance provided to the institution (the institution or group of health care professionals received direct or indirect financial reward or benefit but not tied to being compliant)

2.1.5 Penalty applicable to a health care professional (a health care professional may receive a direct or indirect financial penalty for not complying with a guideline)

2.1.6 Penalty applicable to the institution (the institution or group of health care professionals may receive a direct or indirect financial penalty for not complying with a guideline)

2.1.7 Change in reimbursement (including any addition, subtraction or substitution of a reimbursable product or service that increases the likelihood of improved implementation)

2.1.8 Other

### 2.2 Patients

2.2.1 Incentive applicable to a patient (a patient may receive a direct or indirect financial reward or benefit if provided with care that complies with a guideline)

2.2.2 Grant or allowance provided to a patient (a patient received a direct or indirect financial reward or benefit but not tied to receiving care that complies with a guideline)

2.2.3 Penalty applicable to a patient (a patient may receive a direct or indirect financial penalty if provided with care that does not comply with a guideline)

2.2.4 Other

# 3 Organisational

### 3.1 Health care professionals

3.1.1 Additional human resources provided for implementation (including increase in the number of staff and change in the type and qualifications of staff to facilitate implementation)

3.1.2 Reallocated roles to assist implementation (including any redistribution of roles among health professionals to facilitate implementation)

3.1.3 Creation of an implementation team (including creation of a multidisciplinary team of health professionals who work together on implementation)

3.1.4 Communication between distant health professionals (including establishment of any type of telecommunication link for implementation)

3.1.5 Improved health care professional satisfaction (including any non-financial benefit aimed at improving motivation, commitment and work satisfaction in implementation)

3.1.6 Other

### 3.2 Patients

3.2.1 Consumer participation in governance (including any change in governance that enables patients to recommend the implementation of a guideline)

3.2.2 Consumer feedback, suggestions and complaints (including any new process that uses information from patients to improve implementation)

3.2.3 Other

### 3.3 Structural

3.3.1 Change in organizational structure (including any service reorganization designed to improve implementation)

3.3.2 Change to the setting or site of service delivery (including any translocation of a service designed to improve implementation)

3.3.3 Change in the physical structure, facilities or equipment of a service (including any change to the infrastructure of a service designed to improve implementation)

3.3.4 Change in information & communication technology supporting a service (including any IT application designed and commissioned to improve implementation; e.g. computerized records, patient tracking systems, electronic referral systems, picture archiving and communication system, telehealth system, on-line text messaging)

3.3.5 Change in quality assurance, quality improvement and/or performance measurement systems (including any quality system designed to improve implementation)

3.3.6 Change in the method of service delivery (including any change to how a service is delivered; e.g. replacement of a traditional pharmacy with a mail order pharmacy)

3.3.7 Change in the integration of services (including change in how services are linked and integrated to improve implementation)

3.1.8 Change in risk management provisions (including any change in insurance cover for loss or damage to facilities, injury to staff, adverse patient outcomes and malpractice that encourages implementation)

3.1.9 Other

# 4 Regulatory

4.1 Change in legislation or regulation (include any change which enforces or mandates implementation)

4.2 Change in the ownership or affiliation (include any change which increases the likelihood of implementation success)

4.3 Change in licensing, credentialing or accreditation of the health service and its elements (including any change that is relevant to the status, legality or reputation of the health service and its employees that increases the likelihood of implementation success)

4.4 Other
